# A Lifestyle Behavior Score Predicting Cardiovascular and Other Causes of Death in a Cohort of Middle-Aged Men Followed up Until Extinction

**DOI:** 10.3390/jcdd13080380

**Published:** 2026-08-11

**Authors:** Alessandro Menotti, Paolo Emilio Puddu

**Affiliations:** 1 Association for Cardiac Research, 00182 Rome, Italy; amenotti2@gmail.com; 2 EA 4650—Signalisation, Électrophysiologie et Imagerie des Lésions D’ischémie Reperfusion Myocardique, Normandie Université—UNICAEN, 14000 Caen, France

**Keywords:** lifestyle behaviors, behavior score, mortality, age at death, prediction

## Abstract

**Objective:** To create and test in a population study a behavior score, using as far as possible, procedures based on the *a posteriori* approach. **Material and Methods:** Data from the Italian Rural Areas (IRAs) of the Seven Countries Study of Cardiovascular Diseases (SCS), comprising 1712 middle-aged men enrolled in 1960 and their entry levels of smoking habits, physical fitness and dietary habits, were entered into a Principal Components Analysis, producing an individual overall behavior (factor) score. The end-points were the 62-year follow-up, approaching cohort extinction, mortality for all-cause death and five cause-specific conditions, which formed the dependent variables of a series of Cox models, whose outcome was expressed by hazard ratios for one class increase in the score, and comparing tertile 1 versus tertile 3 of the score, without and with the addition of age, systolic blood pressure, serum cholesterol and high socio-economic status as possible confounders. **Results:** Fatal coronary heart disease (CHD), heart diseases of uncertain etiology (HDUE), stroke, cancer (CAN), chronic bronchitis (CB) and all-cause mortality (ALL) were predicted in a significant way in the four approaches, with a relevant reduction in death rates expressed by hazard ratios ranging from 0.42 to 0.83. Moreover, the influence of the three basic behavior scores showed that CHD was the most strongly associated with them. Age at death for all-cause mortality, on the other hand, was directly related with the full and the three types of behavior score. **Conclusions.** A behavior score based on three common lifestyle habits created by an *a posteriori* approach proved to be significantly associated with various causes of death in a male population group followed-up until extinction.

## 1. Introduction

It has been largely demonstrated that some lifestyle behaviors are strongly related to health and diseases. We mainly refer to smoking [1,2], physical activity [3,4] and dietary habits [5], although other aspects have been investigated. Our research group has produced, in epidemiological investigations, several analyses of this issue, considering one behavior at the time or forcing the three of them into the same multivariate models, mainly to predict mortality [6]; therefore, this aspect can be disregarded.

On the other hand, the literature is relatively rich in contributions that combined different behaviors in various ways in order the look at the problems from different points of view, sometimes creating complex predictive scores [7,8,9,10,11,12,13,14,15,16,17,18,19,20,21,22,23,24,25,26,27,28,29,30]. A clear comparison across these contributions is almost impossible due to the large variations in terms of behavior choices, ways to provide reasonable predictive weights and the different types of final analysis, as we discuss later. Our baseline data were collected 66 years ago, at the beginning of CVD epidemiology and therefore some recent concepts and ideas were not available. The purpose of the present analysis is only an attempt to produce a behavior score combining together smoking habits, usual physical activity and dietary habits derived from a study population of middle-aged men followed-up for mortality during 62 years until practical extinction and to evaluate its capacity to predict several causes of death.

## 2. Material and Methods

Population and Measurements: Data derive from the Italian Rural Areas (IRA) of the Seven Countries Study of Cardiovascular Diseases (SCS), enrolled and first examined in 1960. Out of a roster of 1735 middle-aged men (age range 40 to 59 years), 1712 were examined, with a participation rate of 98.7%.

For the purpose of this analysis, a limited set of risk factors, lifestyle behaviors and personal characteristics were considered as follows:

Additional risk factors, used as confounders in multivariate models, were as follows: (a) age approximated to the nearest birthday (in years); (b) systolic blood pressure measured following the rules given by the WHO Cardiovascular Survey Methods Manual (WHO Manual) [31]; (c) serum cholesterol measured on casual blood sample following the methodology of Anderson and Keys [32]; high socio-economic status, derived by an extensive list of type of work classifications.

Lifestyle behaviors were as follows: (a) Physical Activity—Fitness: Many years ago, physical activity was initially classified into three levels based on the type of profession and a few non-standardized questions. These levels were used in many analyses and showed significant associations, mainly with coronary heart disease (CHD, with vigorous being protective) and also with all-cause mortality. Later, we produced a fitness score (combining into a PCA arm circumference, heart rate and vital capacity) that was shown to be highly correlated with physical activity and showed a stronger predictive power for most end-points [33]. As a consequence, we decided to use fitness as an indicator of physical activity because it is more reliable, being based on measurements. In fact, the association of fitness with physical activity levels was rather strong, with mean levels of fitness of 1.66, 1.79 and 2.12 for sedentary, moderate and vigorous physical activity, respectively (*p* value of ANOVA < 0.0001; power = 1.0000). The individual fitness scores were divided into three equinumerical tertiles arbitrarily called low, intermediate and high, which roughly correspond to the physical activity levels of sedentary, moderate and vigorous. Then, the three tertiles were entered into the PCA that computed the overall behavior score. (b) Smoking habits were derived from a standard questionnaire, classified into three classes (smoker, ex-smoker, never smoker) and later entered in a PCA procedure to produce the behavior score. (c) The basic findings of a dietary survey were 18 food groups that, using a PCA procedure, were used to produce an individual dietary score whose rank list was arbitrarily divided into three equinumerical tertiles. In a large number of published analyses, we found that one of the tertiles included amounts of food groups typical of those of the Mediterranean Diet and was protective (versus the other two tertiles), mainly in relation with CHD and all-cause mortality. As a consequence, the three tertiles were arbitrarily called Mediterranean, Intermediate, and Not Mediterranean, or, as an alternative, Healthy, Moderate, and Unhealthy [34]. Then, the three tertiles were entered into the PCA that computed the overall behavior score [6].

The three tertiles or classes of each behavior underwent another Principal Components Analysis, producing a final behavior score. Subsequently, the final behavior score was subdivided into three equinumerical classes.

Appendix A Table A1 and Table A2 report detailed characteristics of the food groups in the three classes of dietary score and of risk factors in the three classes of fitness. Moreover, the numerical data of some PCA steps producing the overall behavior score are presented in more detail.

End-points were derived from mortality data collected over 62 years after entry examination with the practical extinction of the cohort (1708 deaths, 3 survivors aged 102 to 106 years and 1 lost to follow-up after 50 years at the age of 91 years). Classification of causes of death was made by a single coder, exploiting information from cause of death certificates, interim examinations and special operations on the field, using the 8th Revision of the WHO International Classification of Diseases (ICD-8) [35] and adopting pre-defined criteria. Causes of death included: CHD = coronary heart disease (ICD-8 410, 411, part of 412 and 413, 795); HDUE = heart diseases of uncertain etiology (ICD-8 427, 400, 402–404, part of 412, 414); stroke = cerebrovascular diseases (ICD-8 430–438 (excluding 435); CAN = cancer (ICD-8 140–209); CB = chronic bronchitis and allied conditions (ICD-8 441–443); ALL = all causes of death (ICD-8 000-E999). In case of multiple causes and uncertainties about the first cause, a rank criterium was used, with Violence, Cancer, Coronary Heart Disease, Stroke and others in order. As a side analysis we measured the age at death (AD), a variable that summarizes the health and disease of each individual and provides important information when the study cohort is extinct.

Statistical Analysis: A descriptive analysis was run on the variables described above. Cox proportional hazard models were computed using various causes of death as end-point and as predictors: (a) the behavior score; (b) the behavior score adjusted for the four additional risk factors (age, systolic blood pressure, serum cholesterol, high socio-economic status); (c) the behavior score subdivided in three tertiles; (d) the behavior score subdivided into three tertiles plus the additional risk factors. Findings were expressed as hazards ratio. 

In another series of Cox models, i.e., one for each end-point, behaviors were entered singularly (together with confounders) offering the opportunity to evaluate their role in the predictive power of the six end-points.

The piece of analysis dealing with AD included a linear regression model with AD as the dependent variable and the behavior score as the independent (predictive) variable, and another with the three behavior scores as predictors.

Then, an attempt was made to create a behavior score a priori based on the simple addition of the various components. Specifically, the score was made by adding, for each individual, its rank position (within diet, fitness and smoking) from the worst to the best score, resulting in seven classes with levels from 3 to 9. The expected distribution of events was to find many cases in the lower classes and few cases in the higher ones.

## 3. Results

The risk factor levels reported in Table 1 reflect the situation of a rural environment in the 1960s, with relatively high levels of blood pressure and relatively low levels of serum cholesterol. The prevalence of smokers (embedded into the behavior score) was high, with 61% being smokers. The prevalence of vigorous physical activity was high due to the heavy manual work in the agricultural communities of those times. However, the analysis was run on Fitscore.

The classes of dietary habits were defined in an arbitrary way, but the characteristics of the healthy diet were those of the Mediterranean diet.

The distribution of causes of death suggests a high rate of cancer and a pool of major cardiovascular disease covering about 42% of all-cause mortality, with a relatively low contribution of CHD.

Table 2 reports the percent distribution of the three classes of behavior in the three tertiles of the compacted behavior score. Interestingly enough, in tertile class 1 of the behavior score, 0% of subjects had a high fitness score, 100% were smokers and only 17% of subjects had a healthy diet.

In the behavior score produced by the Principal Components Analysis, high levels correspond to healthier behaviors. As a consequence, in multivariate analysis, an increase in the score corresponded to a reduction in estimated mortality, expressed by a hazard ratio < 1.

Findings related to the Cox model predicting causes of death as a function of behavior score (Table 3) suggest that, in all cases, hazard ratios were lower (more favorable) when the behavior score was forced as a whole, as well as when comparing tertile 1 with tertile 3. In the second case, the hazard ratio is lower than in the first one. In all categories of causes of death, hazard ratios were significant in the four different approaches, with the most favorable results being related to CB. Moreover, in the lower section of the same Table, it is clear that an excess of risk can be found when computing the ratio of tertile 1 to tertile 3 of the score.

In another analytical approach (Table 4) the three individual behavior scores (not compacted into a unique variable) were independently forced in Cox models together with the confounding variables, thus allowing for their impact on the single-morbidity conditions to be observed. Smoking was associated with all six conditions; fitness score was associated with CHD, stroke, CB and all causes; diet score was associated only with CHD and CAN. Among the confounding variables, age was significantly associated with all conditions and high SES with none, with the other results largely agreeing with the expected results.

In a linear regression model, the coefficient of behavior score predicting AD was +2.53, which means that for an increase of one unit in the score, the estimated AD increases by 2.53 years. In this circumstance, the overall mean of the score is practically zero and the standard deviation is 1, while its range is about 3.5 units. In another multiple linear regression model, the coefficients of the three behavior scores (as predictors) were all positive and highly significant.

In the attempt to produce a simple additive score, seven classes were used with levels ranging from 3 to 9. The additive behavior score was made, adding for each individual, its rank position (for diet, fitness and smoking) from the worst to the best score, resulting in seven classes with levels from 3 to 9. The expected distribution of events was to find many cases in the lower classes and few cases in the higher ones. Tests made on some major events showed irregular trends in the seven classes, with p of chi-squared far from significance.

## 4. Discussion

The behavior score proposed in this analysis, may represent an interesting alternative for the evaluation of lifestyle behaviors’ relationship with fatal events. This type of score has the theoretical advantage of being an *a posteriori* approach, that is, it is largely independent from the opinions of the investigators, and thus avoids peculiar (and subjective) choices similar, for example, to those produced by scholars of metabolic syndrome. In fact, its construction is almost entirely driven by a mathematical procedure that initially ignores the end-point of interest and is based only on the inter-correlations of the variables forced into the program. Moreover, two of the variables offered to the system (that is, the dietary score and fitness score) have the same origin, since they derive from the combination of 18 food groups and three personal characteristics, which are then treated by Principal Components Analysis.

We decided to use PCA for the construction of dietary score, fitness score and the overall behavior score in order to rely on a standard programs, avoiding personal choices that are difficult to justify.

Moreover, it is of interest that the factor loadings and coefficients of the PCA provided negative algebraic signs for the sections bound to an adverse effect while the positive ones were associated with the sections carrying beneficial effects; all this was observed in the absence of a known end-point, somewhat anticipating the outcome of the subsequent Cox models, while still being created by a system that ignored the natures of the end-points.

We also attempted to build a simpler score, where predictive weights were assigned to the behavioral variables, giving arbitrary values, and then added up. This empiric score ranged from 3 to 9, and was ultimately unable to discriminate various levels of risk. In any case, we did not aim to present results that could be used for testing different populations since we are aware that the complexity and peculiarity of Principal Components Analysis may prevent easy re-application.

The use of the Cox model in a very long follow-up did not show real failures with a practically extinct cohort. In particular, the Schoenfeld residuals tested against time showed, for all variables, almost circular clouds without any deformation toward the end of the follow-up.

As partly expected from previous experience with this issue, all major groups of causes of death were strongly predicted by the behavior score, with a clear advantage for those with high levels of this score. Dividing the score into three tertiles and comparing tertile 1 with tertile 3 suggested that higher tertiles provide an advantage, while a systematic reduction in the hazard ratios occurred when forcing four extra covariates, representing classical risk factors, into the Cox models. The large predicting power found for CB were likely due to the smoking component of the score. Findings related to all-cause mortality necessarily represented a balanced compromise between causes that were strongly related and causes that were less related to lifestyle behaviors. The choice of the variables that possibly play the role of confounders was limited to age, systolic blood pressure, and serum cholesterol, with the addition of high socio-economic status, which was considered in connection with recent contributions suggesting the need to insert behaviors into the broader idea of social determinants of health [36]. Clearly these concepts did not exist at the beginning of cardiovascular epidemiology in the middle of last century. On the other hand, we did not use body mass index (BMI) as possible confounder, since this variable, in a long series of analyses on this population, never played a significant predictive role except when tested against AD in the extinct cohort and treated in a quadratic way, producing a parabolic relationship [6]. Tests were made adding Body Mass Index as a possible confounder into the Cox models, but this did not influence the findings of the basic variables.

Among the three behaviors, the larger predictive role was played by smoking habits, as shown by the details in Table 4 and in Appendix A Table A2, but the role of dietary habits and fitness were still major contributors. Finally, the strong association of behavior score with AD in this extinct population confirms the interesting role of lifestyle in conditioning longevity.

A limit of our study consists of the small population sample size, which was partially balanced by a follow-up that reached the stage of extinction. Moreover, only male subjects were included. Another common criticism deals with the use of predictive variables measured only at entry examination. However, we have shown in dedicated analyses that risk factors, like serum cholesterol, are significantly predictive up to 40 years. Moreover, the repeated measurements of smoking and dietary habits in the survivors after the first 30 years of follow-up allowed some reasonable conclusions to be reached at least for the role of diet [37]. In particular, the predictive power of baseline dietary habits was strong after the first 30 years and definitely lower from year 30 to year 62. However, the prediction of baseline measurements along the next 62 years, although still lower than for the first 30 years, represented a kind of balance between the observations of the first 30 years and those made with measurements taken at year 30 on survivors and projected to the second block of a 30-year follow-up. Finally, the influence on mortality of the second 30-year time block relied only on a little more than one third of the original cohort, comprising people aged 70 to 89 years [37]. All this suggests that the prediction of events during a very long follow-up is automatically adjusted for intermediate changes in the association with risk factors.

Another limitation is probably due to the peculiar characteristics of the study population, who were located in rural areas in the middle of the last century, with about two thirds being farmers engaged in heavy physical work in the fields, when agriculture mechanization was not yet developed. Moreover, these middle-aged men had a mean daily caloric intake of about 3000 calories, with almost 20% being covered by alcohol of wine-based origin. In addition, leisure physical activity was practically absent in their daily habits. All this may mean that the conclusions have a prevalent historic value and cannot necessarily be applied to other populations with modern characteristics and habits.

The literature is relatively rich in reports on this issue, although the approaches employed in these analyses vary [7,8,9,10,11,12,13,14,15,16,17,18,19,20,21,22,23,24,25,26,27,28,29,30]. Practically all papers used all-cause mortality (and occasionally other derived metrics) as the end-point and had very different durations of follow-up. A majority of them included other end-points, mainly cardiovascular diseases and cancer [9,12,15,17,20,21,24,25,27]. The most commonly used behaviors were smoking habits, physical activity (usually derived from a questionnaire) and dietary habits. However, in many cases alcohol intake was considered an independent behavior [7,18,20,21,22,23,24,25,27]. On the other hand, in rare cases, dietary habits were not considered [7,8]. In rare cases, prediction was based on clearly defined scores [9,10,17,19,24], while at least five papers used a limited number of eating habits (for example, vegetable intake, red meat intake, salt, etc.) and each of them were treated as an independent lifestyle behavior [11,12,15,26,29]. In a majority of cases [7,10,12,13,14,16,18,20,21,22,24,25,27,28], high body mass index, large abdominal circumference and the diagnosis of obesity were considered lifestyle behaviors and not risk factors due to adverse lifestyle behaviors. In a few cases, sleeping habits [8,22,25,28,30] and social interactions [29] were added as extra behaviors.

The associations of the above characteristics with events were constructed in different ways. In a few contributions, each behavior was examined one-by-one [7,8,16,19] or they were simply combined in multivariate models [14,18,28], while in the majority of cases an additive score was created, adding up the number of the behaviors and giving each of them the same weight [9,10,11,12,13,14,15,20,21,22,23,25,26,27,30]. Only in one paper could a real unique score of combined lifestyle behavior be clearly identified [24]. A metanalysis of this issue included studies characterized by several different approaches and it was almost impossible to interpret the conclusions [29].

The literature contains thousands of references, although apparently none related to the role of lifestyle behaviors in extinct-population cohorts. Many of them refer only to psycho-social problems, while others refer to peculiar situations. For example, one paper deals with the integration of environmental and genetic predictors [38]. An interesting contribution describes how a healthy lifestyle may help already aged people to become centenarians [39]. One paper used the so-called life’s essential eight and life’s crucial nine scores to predict mortality in post-menopausal women [40], while another similar paper limited to the use of the life’s essential eight scores predicts major cardiovascular events and total mortality [41]. Also, a large group of subjects with metabolic syndrome were studied for their risk of major non-communicable disease and death as a function of major lifestyle behaviors [42]. Finally, a giant meta-analysis, quoting 146 references, included the most variable types of behavioral characteristics and concluded that these measurable indicators are likely involved in preventing mortality and prolonging life expectancy [43]. Psychological problems and habitual physical activity were among those commonly considered. Despite the limited scientific rigor of many of the above papers, in all of them it was reported that all possible determinants were highly predictive of all-cause mortality or other end-points and were therefore eligible to be considered in the prevention of diseases and promoting longevity.

From this limited review of the current literature on the issue, it appears that our approach is substantially different from the majority of others. We neither classified nor used anthropometric measurements or diagnoses (like BMI, obesity, and girth circumference) as behaviors since they are likely consequences of some behaviors. Moreover, we included alcohol intake in the dietary score because, in our study, population alcohol intake (based on wine) was largely part of meals made more than 60 years ago. Again, we did not use individual parts of dietary habits (say, fruit intake, vegetable intake, red meat intake, etc.) as single behaviors, giving preference to complex dietary scores including all food groups [6]. Our physical activity classification, although based on a questionnaire, was validated by a fitness score and by caloric intake and therefore substituted by a measurable fitness score. Our basic behavior score was created by running a Principal Components Analysis (that is, an *a posteriori* approach) and not by the trivial approach of adding up the various behaviors without a scientific criterium and adopting opinion-based choices like those found in the definition of metabolic syndrome. The dietary score and the fitness score included in our analysis were computed by running a PCA employing another *a posteriori* approach. Despite the small sample size, we had the opportunity to use a cohort followed-up for 62 years until practical extinction and to use AD as an additional end-point.

## 5. Conclusions

The behavior score proposed in this paper showed that: (a) a compact behavior score can confirm findings obtained using separate scores; (b) an analysis based, as far as possible, on *a posteriori* procedures is fully valid to study the association between behaviors, fatal events and age at death; (c) a simple, trivial additive score based on the same data is a rather poor tool. However, our score is not proposed as an alternative to other approaches since it has not been tested on enough different populations.

## Figures and Tables

**Table 1:** Descriptive statistic of variables used in the analysis.

	Additional Risk Factors								
	Mean	SD		Median		25th percentile			75th percentile
Age, years	49.1	5.1		49		45			53
Systolic blood pressure, mmHg	143.6	21.0		140		130			155
Serum cholesterol, mmol/L	5.21	1.06		5.11		4.52			5.79
High socio-economic status, %	11.1	----		----		----			----
	**Lifestyle Behaviors**								
Physical fitness	Low %				Intermediate %			High %	
	33.3				33.3			33.3	
Smoking habits	Smoker %				Ex-smoker %			Never smoker %	
	61.1				13.6			25.4	
Dietary score (tertiles)	Unhealthy %				Intermediate %			Healthy %	
	33.3				33.3			33.3	
			**Death Rates in 62 Years of Follow-Up**						
Cause:			Rate, %				Cumulative rate, %		
CHD			16.4				16.4		
HDUE			12.6				29.0		
Stroke			13.4				42.4		
CAN			27.0				69.4		
CB			3.5				72.9		
Other			26.9				99.8		
ALL			99.8						

CHD = coronary heart disease; HDUE = heart diseases of uncertain etiology; stroke = cerebrovascular diseases; CAN = cancer; CB = chronic bronchitis and allied conditions; ALL = all causes of death.

**Table 2:** Distribution of three behaviors (%) into three behavior score classes.

	Behavior Score Tertile 1	Behavior Score Tertile 2	Behavior Score Tertile 3
**Physical Fitness**			
Low	63	17	20
Intermediate	37	26	37
High	0	57	43
Total	100	100	100
**Smoking habits**			
Smoker	100	83	0
Ex-smoker	0	6	35
Never smoker	0	11	65
Total	100	100	100
**Dietary score**			
Unhealthy	46	33	22
Intermediate	37	23	40
Healthy	17	44	38
Total	100	100	100

**Table 3:** Findings of Cox models using behavior score, as a whole or divided into three tertiles, to predict various causes of death.

End-Point	Full Behavior Score		Adjusted Full Behavior Score	
	Hazard ratio	95% CI	Hazard ratio	95% CI
CHD	0.75 *	0.65–0.87	0.78 *	0.68–0.91
HDUE	0.81 *	0.68–0.96	0.82 *	0.70–0.98
Stroke	0.79 *	0.67–0.93	0.83 *	0.71–0.97
CAN	0.73 *	0.66–0.82	0.74 *	0.66–0.82
CB	0.42 *	0.29–0.59	0.42 *	0.29–0.60
ALL	0.76 *	0.71–0.81	0.78 *	0.74–0.83
	**Tertile 1 versus Tertile 3** **of Behavior Score**		**Tertile 1 versus Tertile 3** **of Adjusted Behavior Score**	
	Hazard ratio	95% CI	Hazard ratio	95% CI
CHD	1.76 *	1.32–2.35	1.63 *	1.22–2.17
HDUE	1.51 *	1.10–2.07	1.43 *	1.00–2.05
Stroke	1.61 *	1.17–2.22	1.45 *	1.05–2.01
CAN	1.85 *	1.47–2.33	1.83 *	1.15–2.31
CB	5.04 *	2.50–10.03	5.00 *	2.50–10.00
ALL	1.71 *	1.54–1.95	1.63 *	1.45–1.84

In the upper part of the table, numerical data report hazard ratios (a) for the increase in the behavior score of one unit in the case of the full score; (b) for the ratio of tertile 1 (active) versus tertile 3 (reference) of the behavior score. Estimates report data not adjusted and adjusted for the four factors (age, systolic blood pressure, serum cholesterol and high socioeconomic status). In the lower part of the table, the hazard ratios show the outcome for a one-unit decrease in behavior score. CI: confidence interval; CHD = coronary heart disease; HDUE = heart diseases of uncertain etiology; stroke = cerebrovascular diseases; CAN = cancer; CB = chronic bronchitis and allied conditions; ALL = all causes of death. * Significant with *p* ≤ 0.05. Unexpectedly, the hazard ratios of models adjusted for the four potentially cofounding variables are not substantially different from those produced by the not-adjusted models.

**Table 4:** Cox models with the three behaviors as single variables plus confounders, predicting six different causes of death.

CHD	Age	SES	SBP	CHOL	SMOKE	FITNESS	DIET
Coeff	0.0676	−0.1067	0.0145	0.0059	−0.1614	−0.1868	−0.2054
P	<0.0001	0.5674	<0.0001	<0.0001	0.0217	0.0174	0.0098
HR	1.40	0.90	1.34	1.27	0.85	0.83	0.81
CI	1.23–1.60	0.62–1.30	1.18–1.51	1.13–1.42	0.74–0.98	0.71–0.97	0.70–0.95
**HDUE**							
Coeff	0.1808	−0.0039	0.0148	0.0009	−0.2795	0.0483	0.1505
P	<0.0001	0.9867	0.0002	0.5998	0.0006	0.6035	0.0991
HR	2.47	1.00	1.34	1.04	0.76	1.05	1.16
CI	2.09–2.92	0.63–1.58	1.15–1.57	0.90–1.19	0.64–0.89	0.87–1.26	0.97–1.39
**Stroke**							
Coeff	0.1187	0.2809	0.0140	0.0033	−0.1731	−0.1831	0.0807
P	<0.0001	0.1407	<0.0001	0.0447	0.0248	0.0371	0.3544
HR	1.81	1.32	1.32	1.14	0.84	0.83	1.08
CI	1.56–2.10	0.91–1.92	1.15–1.52	1.00–1.30	0.72–0.98	0.70–0.99	0.91–1.29
**CAN**							
Coeff	0.0807	−0.2425	0.0013	0.0016	−0.2512	−0.0982	−0.1735
P	<0.0001	0.1358	0.6405	0.1737	<0.0001	0.1093	0.0045
HR	1.50	0.78	1.03	1.07	0.78	0.91	0.84
CI	1.35–1.66	0.57–1.08	0.92–1.14	0.97–1.17	0.70–0.87	0.80–1.02	0.75–0.95
**CB**							
Coeff	0.1394	0.1425	−0.0174	−0.0088	−0.5231	−0.9317	−0.0599
P	<0.0001	0.7159	0.0431	0.0155	0.0032	<0.0001	0.7233
HR	2.01	1.15	0.71	0.70	0.59	0.39	0.94
CI	1.49–2.71	0.54–2.48	0.50–0.99	0.53–0.93	0.42–0.84	0.27–0.57	0.68–1.31
**ALL**							
Coeff	0.1036	−0.0766	0.0107	0.0021	−0.1941	−0.1680	−0.0491
P	<0.0001	0.3341	<0.0001	0.0006	<0.0001	<0.0001	0.1247
HR	1.68	0.93	1.24	1.09	0.82	0.85	0.95
CI	1.59–1.77	0.79–1.08	1.17–1.30	1.04–1.14	0.78–0.87	0.79–0.90	0.89–1.01

CHD = coronary heart disease; HDUE = heart diseases of uncertain etiology; stroke = cerebrovascular diseases; CAN = cancer; CB = chronic bronchitis; ALL = all-cause mortality; Coeff = coefficient; P = probability; HR = hazard ratio; CI = 95% confidence intervals. Brackets show the units used for the estimate of HR: age = age (5); SES = high socio-economic status (1); SBP = systolic blood pressure (20); CHOL = serum cholesterol (1); SMOKE = smoking score (1); FIT = physical fitness score (1); DIET = dietary score (1). HR derives from the increase in units given above for each variable.

## Data Availability

The data and computing codes are not available for replication because the original data are not publicly available, although the Board of Directors of the Study may evaluate specific requests for dedicated analyses.

## Appendix A

**Table A1:** Details of the components of the three behavior scores.

	Components Of Dietary Score in Grams per 1000 Calories/Day			
Food Group	Unhealthy Diet	Moderate Diet	Healthy Diet	*p* of ANOVA
Bread	93.5 (41.4)	122.2 (58.3)	146.7 (65.5)	<0.0001 (*)
Cereals	40.8 (20.4)	41.2 (19.0)	42.8 (19.7)	0.1773
Potatoes	7.2 (7.7)	8.3 (8.7)	9.8 (9.1)	<0.0001 (*)
Vegetables	15.6 (15.5)	17.1 (16.1)	24.5 (19.6)	<0.0001 (*)
Legumes	1.7 (6.5)	1.5 (2.9)	1.2 (2.6)	0.1404
Fruit	93.5 (59.4)	68.8 (49.9)	32.8 (38.2)	<0.0001
Oils	13.4 (6.9)	14.1 (7.0)	13.5 (6.4)	0.1261
Meat	50.7 (26.6)	43.8 (23.9)	27.8 (18.2)	<0.0001 (*)
Fish	6.7 (6.4)	7.0 (6.5)	9.2 (8.4)	<0.0001 (*)
Eggs	5.1 (6.0)	5.9 (7.9)	6.9 (7.9)	0.0003
Butter	7.0 (6.2)	4.6 (5.0)	0.7 (2.0)	<0.0001 (*)
Margarine	0.6 (1.7)	4.1 (4.8)	0.7 (1.9)	0.2294
Milk, solid part	7.7 (7.1)	4.1 (4.8)	0.7 (1.9)	<0.0001 (*)
Cheese	7.4 (5.2)	4.8 (8.0)	3.2 (5.9)	<0.0001 (*)
Sugar	7.4 (5.2)	4.7 (3.5)	1.8 (2.7)	<0.0001 (*)
Pastries	7.8 (12.2)	4.0 (4.8)	1.5 (2.8)	<0.0001 (*)
Alcohol	23.1 (15.4)	28.7 (18.3)	29.0 (20.7)	<0.0001
Sugar beverages, sugar	0.4 (1.8)	0.1 (0.7)	0.04 (0.2)	<0.0001 (*)
(*) Cases where the significant differences between healthy diet and unhealthy diet suggest a Mediterranean characteristic of the healthy diet.				
	**Components Of Fitness Score**			
	Low fitness	Intermediate fitness	High fitness	*p* of ANOVA
Arm circumference, mm	256	268	282	<0.0001
Heart rate, beats/min	81.3	69.2	63.4	<0.0001
Vital Capacity, L/m squared	1.45	1.65	1.84	<0.0001
	**Components Of Smoking Score**			
	Smoker	Ex-smoker	Never Smoker	*p* of chi squared
N	1046	232	434	<0.0001
%	61.1	13.6	25.4	

**Table A2:** Some numerical characteristics of the nine components entered in the PCA for the computation of overall behavior score.

Components	Factor Loading After Varimax Rotation	Factor Coefficients After Varimax Rotation
Unhealthy diet	−0.3484	−0.1910
Intermediate diet	0.0726	0.0398
Healthy diet	0.2758	0.1512
Low fitness	−0.3971	−0.2177
Intermediate fitness	−0.0230	−0.0162
High fitness	0.4266	0.2339
Smoker	−0.8459	−0.4637
Ex-smoker	0.3800	0.2083
Current smoker	0.6489	0.3557

Note. Following the estimates of the eigenvalues, six of the above variables captured more than 100% of the variance.
